# Is the flap reinforcement of the bronchial stump really necessary to prevent bronchial fistula?

**DOI:** 10.1186/s13019-020-01290-0

**Published:** 2020-09-11

**Authors:** Fatmir Caushi, Gentiana Qirjako, Ilir Skenduli, Daniela Xhemalaj, Hasan Hafizi, Silva Bala, Alban Hatibi, Arian Mezini

**Affiliations:** 1 Department of Thoracic Surgery, University Hospital “Shefqet Ndroqi”, Tirana, Albania; 2 Department of Surgery, Our Lady of Good Counsel University, Tirana, Albania; 3 Department of Public Health, University of Medicine, Tirana, Albania

**Keywords:** Bronchial fistula, Bronchial stump, Flap reinforcement, Lung resection, Complication

## Abstract

**Background/aim:**

The development of bronchopleural fistula (BPF) remains the most severe complication of lung resection, especially after pneumonectomy. Studies provide controversial reports regarding the benefits of flap reinforcement of the bronchial stump (FRBS) in preventing BPF’s occurrence.

**Methods:**

This is a retrospective cohort study of 558 patients that underwent lung resection in a 12-year period (from 2007 to 2018). Ninety patients (16.1%) underwent pneumonectomy. Patient follow-up period varied from 1 to 12 years.

**Results:**

Out of 558 patients in this study, 468 (83.9%) underwent lobectomy, and the remnant underwent pneumonectomy. In 114 cases with lobectomy, only 24.4% had FRBS, meanwhile in 56 cases with pneumonectomy only 62.2% had FRBS. BPF occurred in 8 patients with lobectomy (1.7%) and in 10 patients with pneumonectomy (11.1%). Among cases with post-pneumonectomy BPF, 6 (10.7%) had FRBS performed, while no FRBS was performed among patients with post-lobectomy BPF, although these data weren’t statistically (*p* > 0.05). In 24 patients (20 lobectomies and 4 pneumonectomies) with lung cancer (10.4%) neoadjuvant treatment was performed, in which 20 patients underwent chemotherapy and 4 underwent radiotherapy. FRBS was applied in each of the above 24 operative cases, but only in 4 of them the BPF was verified.

**Conclusion:**

The idea of enhancing the blood supply through the FRBS for BPF prevention has gain traction. Although FRBS has been identified as valuable and effective method in BPF prevention following lung resection, our study results did not support this evidence.

## Introduction

Lung resection remains the treatment of choice for bronchogenic carcinoma and intractable end-stage localized lung disease such as tuberculosis, bronchiectasis, lung abscess and hydatidosis. Nonetheless, lung resection is a risk in itself for post-operative complications, which accounts for significant morbidity and mortality rates [1–3]. The development of bronchopleural fistula (BPF) remains the most severe complication that may arise after lung resection, especially after pneumonectomy which is complicated with persistent empyema, aspiration of fluid from pleural cavity, or pneumonia of the remaining lung. In the last decade, significant improvement in surgical techniques, antibiotic therapy, and postoperative care have led to a decrease in BPF incidence post-pneumonectomy from 28% to about 4% [3, 4].

Intra-operative techniques to prevent BPF include minimizing dissection, a short bronchial stump, bronchial stump closure with staplers rather than suture, or flap reinforcement of the stump with surrounding tissues such as pleura, intercostal muscle, pericardial fat pad, or diaphragm and azygous vein.

The role of the flap reinforcement of the bronchial stump (FRBS) is to enhance the blood supply to the level of the bronchial stump, thus preventing the dehiscence of the stump. Surgical techniques that use flap reinforcement appear to play an important role in the prevention of bronchial fistula, especially after neo-adjuvant chemotherapy [1, 3, 5, 6]. There have been many studies that compared effectiveness of different types of flaps in preventing BPF. Berna et al. suggested bronchial stump reinforcement using a thoracodorsal artery perforator flap [7], while Mineo and Ambrogi found that diaphragmatic flap was practical, safe and useful for suture line protection or treatment of BPF [8].

Abolhoda et al. reported that use of the latissimus dorsi flap to seal the bronchial stump was also feasible [9]. D’Andrilli et al. concluded that omental flap transposition is effective at prevention and treatment of post-pneumonectomy BPF [10].

Pericardial flaps also have been found feasible, safe and effective without increasing operative time as reported by Hamad et al. [11]

Although some studies favor the use of flap to cover the stump for BPF prevention [12], others suggest otherwise [2]. We have used FRBS various times in our practice, especially in cases of neoadjuvant treatment or post-pneumonectomy. However, BPF is seen even after flap reinforcement of the bronchial stump, making the role of FRBS questionable. Our current study aims to investigate further the usefulness of covering the bronchial stump with a flap to prevent BPF occurrence.

## Materials and methods

This is a retrospective cohort study of 558 patients that underwent lung resection in a 12-year period (from 2007 to 2018). Out of participating patients, 90 (16.3%) underwent pneumonectomy. Patient follow-up period varied from 1 to 12 years.

We performed standardized pre-operative diagnostic work-up for all the patients, consisting in chest X-ray P/A and lateral views, enhanced computed tomography (CT), fiberoptic bronchoscopy, pulmonary function test (PFT), cardiac evaluation by ECG and full laboratory investigation.

All patients were operated under general anesthesia and using a standard postero-lateral thoracotomy. In some patients, the fifth intercostal muscle was prepared to use as flap. After the mobilization of the lung from the chest wall, the pulmonary artery (or its branches) and veins were dissected and divided (depending on the grade and part of resection). In both sides the bronchus was freed from the surrounding lung tissue, and then divided and closed cartilage to membranous by running sutures of 3\0 vicryl (2 rows) or by stapling technique with Endo GIA 45 mm Extra Thick (when it was possible).

Airtight seal test of the closed bronchial stump under saline solution and increased airway pressure was performed in all patients. In 170 patients (30.5%), the flap (intercostalmuscle, pericardial fat pad, pleura) was turned over the bronchial stump and fixed to the peribronchial tissues by interrupted 3/0 vicryl sutures. In 94 patients (55%) we used intercostal muscle as the flap, in 48 patients (28%) pericardial fat pad, and in 28 patients (17%) pleura.

FRBS was generally performed in cases considered to be at high risk of BPF, particularly after pneumonectomy, either in cases that had undergone neoadjuvant treatment or were candidates for adjuvant treatment. However, the decision to execute FRBS was made by the surgeon in the operating room.

In patients with symptoms suggesting BPF, a CT-scan was performed, but ultimately a flexible bronchoscopy was decisive in establishing the final diagnosis. Patients diagnosed with BPF were treated surgically with a simple chest drain or with the “open window” technique, especially in post-pneumonectomy cases.

Chi-square and/or Fisher’s exact test were used to compare the distribution of cases with FRBS and fistula across both types of intervention (lobectomy vs. pneumonectomy). Binary logistic regression was used to assess the presence of clinical characteristics and treatment of patients (chemiotherapy and radiotherapy) as predictor variables and fistula (outcome variable) among survey participants. Crude (unadjusted) odds ratios (ORs: non-fistula vs. fistula), their respective 95% confidence intervals (95%CIs) and *p*-values were calculated. All statistical analysis was performed using SPSS (Statistical Package for Social Sciences), version 17.0.

## Results

Our study included 558 patients, among whom, 14.4% were females and 85.6% males. Average age was 62.4 years (±12SD). The main diagnosis in 433 cases (77.6%) was lung cancer (According to TNM classification, 10.2% of cases were Stage I, 44% Stage II, 21.3% Stage III, and 5.6% Stage IV) and in 125 patients the main diagnoses were: echinococcus of the lung, bronchiectasis, lung abscess, tuberculosis and hemoptysis. (Table 1.) Most of patients, 468 (83.9%), underwent lobectomy, while 90 patients (16.1%) underwent pneumonectomy (Table 1). In only 106 patients (90 lobectomies and 16 pneumonectomies) (18.9%), the bronchial stump was closed by stapler.

**Table 1:** Description of the study population

Variables	N (%)
**Sex**	
Male	478 (85.6%)
Female	80 (14.4%)
**Mean age**	62.4 ± 12 SD yrs
**Diagnosis**	
Lung cancer	433 (77.6%)
Lung Echinoccocus	36 (6.5%)
Bronchiectasis,	34 (6.1%)
Lung abscess,	33 (5.9%)
Tuberculosis	22 (3.9%)
**Type of intervent**	
Lobectomy	468 (83.9%)
Pneumonectomy	90 (16.1%)
**Complications**	
No	463 (82.9%)
Yes	95 (17.1%)

**Fig. 1:**
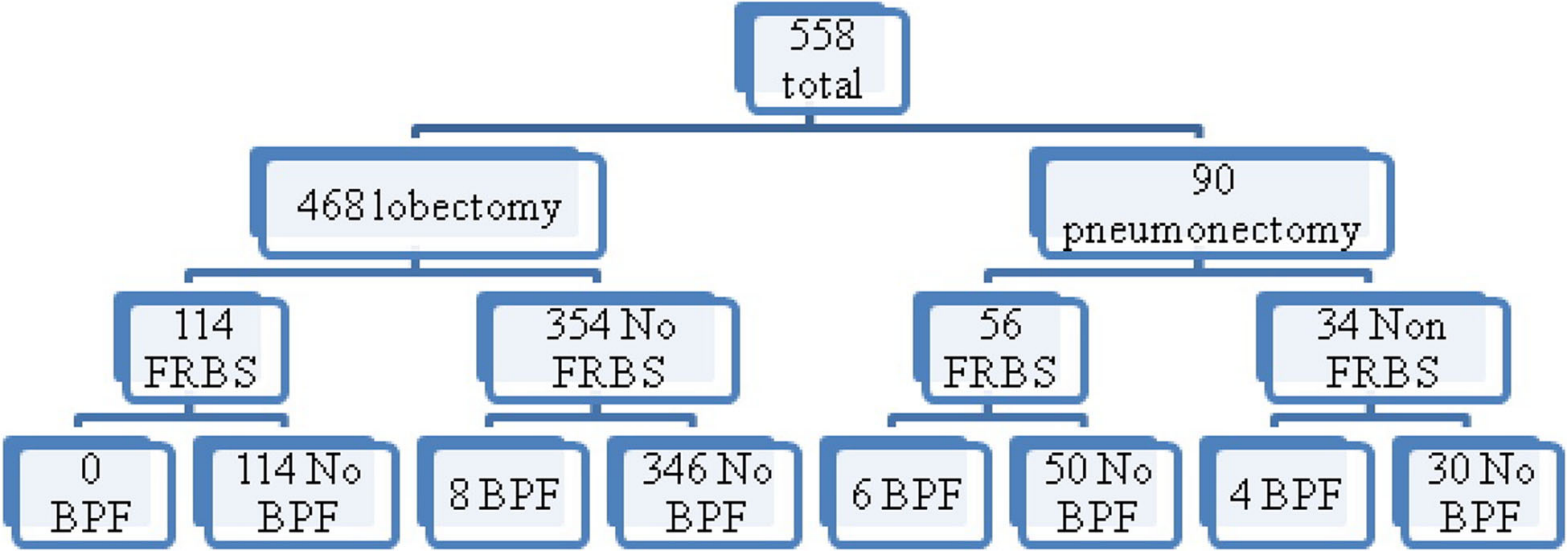
Distribution of cases regarding the type of resection and BPF presence

Among all study patients, 95 (17.0%) presented complications after the surgery, and 18 (3.2%) developed BPF, with a postoperative 30-day mortality rate of 3%. Causes of death were severe pneumonia and heart attack but no patient died because of bronchial fistula. Six patients (4 patients undergoing pneumonectomy and 2 lobectomy) developed BPF in the first 2 weeks after the surgery, and 12 patients developed it 4 months after the surgery. Three out of 4 patients who developed BPF after pneumonectomy in the first 2 weeks had FRBS. Only two cases (one patient after lobectomy and one after pneumonectomy) had a complete failure of the bronchial stump. Post-lobectomy BPF occurred in eight patients (1.7%), while post-pneumonectomy BPF happened in ten (11.1%) patients **(**Fig. 1**)**. BPF was observed in 3.7% of the patients operated for lung cancer and only in 1.6% of the patients operated for other lung pathologies. The BPF prevalence was significantly higher among patients with pneumonectomy compared to those with lobectomy (11.1% vs. 1.7%: Fisher exact test: *P* = 0.007) (Table 2). Among 10 patients that developed post-pneumonectomy BPF, only four of them underwent right pneumonectomy, while among patients with post-lobectomy BPF, 75% of them had lower lobectomy.

**Table 2:** Flap reinforcement of the bronchial stump and broncho-pleural fistula presence by types of lung resection

	Lobectomy (***N*** = 468)* N (%)	Pneumonectomy (***N*** = 90)* N (%)	***P*** value
Flap reinforcement			
Yes	114 (24.4)	56 (62.2)	*P* < 0.001
No	354 (75.6)	34 (37.8)	
Presence of BPF			
Yes	8 (1.7)	10 (11.1)	*P* = 0.007
No	460 (98.3)	80 (88.9)	

* Number of cases and column percentages (in parenthesis)

FRBS was performed in 170 patients (30.5% of all study cases), 114 post-lobectomy (24.4%) and 56 post-pneumonectomy (62.2%). Tables 3 and 4 depict the correlation between FRBS and BPF occurrence across types of surgery (pneumonectomy or lobectomy).

**Table 3:** Post-pneumonectomy BPF by flap reinforcement of the bronchial stump procedure

	Yes (***N*** = 56)*	No (***N*** = 34)*	***P*** value
**Presence of BPF**			
Yes	6 (10.7)	4 (11.8)	*P* = 0.6
No	50 (89.3)	30 (88.2)	

*Number of cases and column percentages (in parenthesis)

**Table 4:** Post-lobectomy BPF by flap reinforcement of the bronchial stump at patients’ procedure

	Yes (***N*** = 114)*	No (***N*** = 354)*	***P*** value
**Presence of BPF**			
Yes	0 (0)	8 (2.3)	*P* = 0.58
No	114 (100)	346 (97.7)	

*Number of cases and column percentages (in parenthesis)

Post-pneumonectomy BPF occurred in 10.7% of the patients that underwent FRBS, versus 11.7% among patients without the FRBS procedure. On the other hand, post-lobectomy BPF did not occur in any of the patients who had the FRBS procedure compared to 2.3% among patients who did not have the FRBS, although the differences were not statistically significant (*p* > 0.05; Fisher’s exact test).

There was not seen any correlation between the type of flap and the incidence of BPF (*P* = 0.419; Chi-square test).

Twenty-four patients (20 lobectomies and 4 pneumonectomies) with lung cancer (10.4%) underwent neoadjuvant treatment, among whom 20 underwent chemotherapy and four underwent radiotherapy. FRBS procedure was performed in all 24 patients, but BPF was diagnosed only among all four pneumonectomy cases.

We found the BPF rate to be greater among patients over 60 years of age and who were heavy smokers [13] and heavy alcohol users (according to Centers of Disease and Control and Prevention) and this result was statistically significant as shown in Table 5.

**Table 5:** Distribution of cases with BPF related to risk factors and concomitant diseases

	Presence of fistula		***P***-value
	Yes (***N*** = 18)^**a**^	No (***N*** = 540)	
**Age**			
< 60 years old	0 (0)	214 (39.6)	***P*** **= 0.014**
≥ 60 years fold	18 (100)	326 (40.4)	
**Gender**			
Male	12 (66.7)	366 (67.8)	***P*** **= 0.04**
Female	6 (33.3)	174 (32.2)	
**Smoker**			
No	18 (100)	196 (36.3)	***P*** **= 0.02**
Yes	0 (0)	344 (63.7)	
**Alcohol user**			
Yes	18 (100)	192 (36.2)	***P*** **< 0.001**
No	0 (0)	338 (63.8)	
**Concomitant diseases**			
Without concomitant diseases	2 (11.1)	282 (52.2)	
Cardio-Vascular disease	12 (66.7)	134 (24.8)	
Other pulmonary diseases	4 (22.2)	52 (9.6)	
Infective pulmonary disease	0 (0)	18 (3.3)	***P*** **= 0.7**
Diabetes Mellitus	0 (0)	14 (2.6)	
Other lung malignancies	0 (0)	14 (2.6)	
Renal diseases	0 (0)	4 (0.7)	
Hemoptyzis	0 (0)	2 (0.4)	
Metastasis	0 (0)	2 (0.4)	
Other diseases	0 (0)	18 (3.3)	

^a^Number of cases and column percentages (in parenthesis)

We found a higher rate of BPF among patients with cardiovascular and other pulmonary diseases compared to those with other concomitant illnesses, but this result was not statistically significant (Chi-square test) (Table 5). Regarding BPF treatment, 12 cases (70%) were treated by performing an “open window” procedure while others were treated by drainage.

Table 6 presents estimates from crude binary logistic regression (unadjusted) analysis. In unadjusted model, the presence of fistula is significantly associated with the presence of diabetes (OR = 1.92, 95% CI = 1.04–2.98), infective pulmonary disease (OR = 2.06, 95% CI = 1.67–3.58), and other pulmonary diseases (OR = 1.86, 95% CI = 1.44–2.99) but not with cardiovascular diseases (OR = 0.91, 95% CI = 0.90–1.11) and malignancies (OR = 1.02, 95% CI = 1.01–1.66).

**Table 6:** Association of Fistula with the presence of some of the concomitant diseases of the study participants; (unadjusted results from binary logistic regression)

Variable	OR*	95%CI*	***P***-Value*
**Diabetes Mellitus:**			
No	1.00	Reference	0.026
Yes	1.92	1.04–2.98	
**Cardio-Vascular disease:**			
No	1.00	Reference	0.102
Yes	0.91	0.90–1.11	
**Infective Pulmonary diseases:**			
No	1.00	Reference	
Yes	2.06	1.67–3.58	0.019
**Other Pulmonary diseases:**			
No	1.00	Reference	
Yes	1.86	1.44–2.99	0.044
**Malignancies:**			
No	1.00	Reference	
Yes	1.02	1.01–1.66	0.102

*Odds ratio (OR: fistula yes vs. fistula no), 95%CIs and *P*- value from unadjusted Binary Logistic Regression

On the other hand, Table 7 presents association of fistula with neoadjuvant treatment (chemotherapy and radiotherapy) between survey patients. In this unadjusted model, the presence of fistula is highly associated with radiotherapy (OR = 2.99, 95% CI = 2.20–6.12), and chemotherapy (OR = 2.11, 95% CI = 1.99–4.11).

**Table 7:** Association of Fistula with chemotherapy and radiotherapy of the study participants; (unadjusted results from binary logistic regression)

Variable	OR*	95%CI*	***P***-Value*
**Chemotherapy:**			
No	1.00	Reference	0.019
Yes	2.11	1.99–4.11	
**Radiotherapy:**			
No	1.00	Reference	0.006
Yes	2.99	2.20–6.12	

* Odds ratio (OR: fistula yes vs. fistula no), 95%CIs and P- value from unadjusted Binary Logistic Regression

## Discussion

BPF is a serious complication of lung resection that leads to persistent empyema, aspiration of fluid from pleural cavity, and pneumonia of the remaining lung. Previous studies have identified an array of risk factors for bronchopleural fistula, which can be categorized as major and minor [1, 2].

Major risk factors include: a) Technical errors, generally during hand-sewing of bronchial stump, b) Untreated lung infection during preoperative phase or postoperative empyema, c) Devascularization of bronchial stump following aggressive mediastinal lymph nodes dissection, or excessive use of electrosurgery for hemostasis, and d) Creation of an excessively long bronchial stump. In a study of 242 consecutive patients, Algar et al. found that long bronchial stumps increased BPF risk [3]. Other major risk factors highlighted in previous studies include: e) pre-operative chemotherapy and irradiation [1, 3, 5], f) use of steroids, g) older age and malnutrition, h) postoperative mechanical ventilation [1–3] and i) residual tumor at bronchial stump. Lindner et al., in a retrospective cohort study of 243 patients, followed over a seven-year period, concluded that advanced tumor stage and carcinomatous lymphangiosis at resection margin, increased the incidence of BPF [6].

A fistula is more likely in cases where there is low pre-operative forced expiratory volume (FEV1) and diffusing capacity of the lung for carbon monoxide (DLCO), increased intravenous fluid in the first 12 h, and after blood transfusions. Algar et al. showed that postoperative mechanical ventilation and chronic obstructive lung disease (COPD) were also independent predictors of BPF. Increased predicted postoperative FEV1 showed a trend towards some protective effect [3].

In our study of 558 patients who underwent pulmonary resection (pneumonectomy performed in 90 patients), the use of FRBS did not have significant value in the prevention of BPF. However, based on our study results and findings from other publications [2] on this topic, it would be incorrect to conclude that FRBS is not a useful procedure in preventing BPF. Our study has several limitations. The higher BPF incidence, found in our study, among patients with pneumonectomy (11.1%) compared to those with lobectomy (1.7%), confirms previous findings that pneumonectomy carries higher risk for BPF development compared to lobectomy [14]. As described the fistula is commonly found on the stump due to the increased risk of ischemic necrosis or pooling of secretions leading to bacterial overgrowth and colonization. The increased risk of BPF associated with pneumonectomy is due to extensive lung resection [14]. The high incidence of BPF after neoadjuvant therapy (17%) may have to do with the quality of neoadjuvant treatment [1, 3, 5].

Among documented risk factors and concomitant diseases, it appears that the use of alcohol, smoking and cardiovascular disease or other lung diseases is associated with BPF development after lung resection, as reported in other publications [1–3]. However, only the associations between BPF and heavy smoking and drinking were statistically significant in our study. Many studies have questioned the role of flaps in preventing BPF, regardless the type of flap used. Taghavi et al. in a retrospective study of 93 patients who underwent pneumonectomy for primary lung cancer, all using a pedicled pericardial flap for bronchial stump coverage, identified no BPF during follow-up [15].

Sfyridis et al., in a randomized controlled trial that included 70 patients from a single center who had lung malignancy, concluded that there was lower incidence of BPF among patients in whom intercostal muscle flap was used compared to control (IF) (0% vs 8.8%, respectively *P* = 0.02). IF in diabetic patients decrease the incidence of both BPF and empyema compared to the use of conventional closure alone [16].

Klepetko et al., on the other hand, analyzed retrospectively 129 patients who underwent pneumonectomy during a 10-year period and concluded that coverage of the bronchial stump did not reduce the incidence of BPF [12].

Also, Deschamps et al., in a retrospective analysis of 713 patients undergoing pneumonectomy, over a 13-year period at a single center, concluded that the reinforcement of the stump was associated with a higher unadjusted risk of both BPF and empyema. Suture of the bronchial stump alone did increase the risk of BPF compared with stapled closure [1]. It is possible that the technique was performed in high-risk patients, yielding worse outcomes.

Algar et al. concluded that the absence of bronchial stump coverage was an independent predictor of BPF in the final multivariable model (RR 1.65 uncovered vs covered, *P* = 0.039). Several FRBS techniques were used, among which intercostal muscle and mediastinal fat pads were the most used [2].

In order to evaluate BPF risk among patients receiving post-pneumonectomy FRBS with any tissue versus among those that did not, Di Maio et al. performed a meta-analysis of studies published in PubMed between 1999 and 2012. The authors concluded that BPF incidence in patients considered as high risk and receiving bronchial stump coverage was only slightly higher compared with patients considered at low risk and not undergoing the procedure. A randomized trial would help answer the question [2].

Our study has various limitations. The cases selected for flap reinforcement were generally at greater risk for developing BPF. We chose to perform FRBS in all patients who had undergone neoadjuvant treatment (20 patients after chemotherapy and 4 after radiotherapy) and/or should be candidates for cancer treatment in the future (stages III and IV according to TNM classification). At the same time, all patients, suffering from a concomitant disease such as cardiovascular disease, COPD, diabetes mellitus or lung infection, received the FBRS procedure. A randomized trial would have been better suited to respond to the study question. Secondly, in some of the cases, an improper collection method of the flap was used, which can result in a devascularized flap. However, this apparently subjective shortage had a very limited impact on the results of this study because the technique of flap harvesting has been standardized. Thirdly, number of cases with pneumonectomy was not very large, only 90 patients. However, the number of patients with lobectomy (468 patients) was large enough to add more precision to the findings in this group. Last, it seems that it is a heterogeneous population but especially in cases of pneumonectomy only two patients had a non malignant disease that could not interfere so much at the results of this study. Of note, in our daily practice we achieved to define standardized interventional techniques regardless of the pathological nature. Therefore in this case the need for the use of FRBS should not be considered as a discriminating procedure in lung resections for malignant tumors compared to that performed for non-neoplasms.

## Conclusion

The development BPF is still one of the most severe complications that may occur after lung resection and this study confirmed that the BPF prevalence was significantly higher among patients with pneumonectomy compared to those with lobectomy.

The idea of enhancing the blood supply through the FRBS post lung resection is important for BPF prevention. Use of intercostal muscle flap, pericardial fat pad and pleural flap are reported as valuable and effective methods in prevention of post-lung resection BPF, especially in pneumonectomies and broncho-sleeve lung resections. However, our study did not find a statistically significant positive effect of the use of flaps in reduction of BPF rate.

## Data Availability

The datasets used and/or analyzed during the current study are available from the corresponding author on reasonable request.
